# Localized co-delivery of collagenase and trastuzumab by thermosensitive hydrogels for enhanced antitumor efficacy in human breast xenograft

**DOI:** 10.1080/10717544.2018.1474971

**Published:** 2018-06-26

**Authors:** Anni Pan, Zhaoyang Wang, Binlong Chen, Wenbing Dai, Hua Zhang, Bing He, Xueqing Wang, Yiguang Wang, Qiang Zhang

**Affiliations:** a State Key Laboratory of Natural and Biomimetic Drugs, School of Pharmaceutical Sciences, Peking University, Beijing, China;; b Beijing Key Laboratory of Molecular Pharmaceutics, School of Pharmaceutical Sciences, Peking University, Beijing, China

**Keywords:** Thermosensitive hydrogel, trastuzumab, collagenase, sustained release, antitumor efficacy

## Abstract

Modulation of the collagen-rich extracellular matrix (ECM) in solid tumors by the treatment with collagenase has been proved effective in enhancement of the interstitial transport and antitumor efficacy of antibodies. We, therefore, developed a PLGA-PEG-PLGA polymer-based thermosensitive hydrogel, which incorporated a HER2-targeted monoclonal antibody trastuzumab and collagenase (Col/Tra/Gel) for peritumoral administration. HER2-positvie BT474 tumor-bearing mice were selected as a model. The Col/Tra/Gel showed the continuous and biphasic release of protein drugs for 9 days *in vitro*. NIR imaging studies demonstrated a long-term retention of Col/Tra/Gel hydrogel in the peritumoral area for over 20 days. Treatment with Col/Tra/Gel reduced the collagen density and enhanced apoptotic cell death in tumor tissue, resulting in superior treatments with increased efficacy and reduced toxicity compared with other control groups. Moreover, a quarter-dose of Col/Tra/Gel exhibited a better antitumor efficacy than that of intravenous injection of clinical trastuzumab formulation. This localized co-delivery system offers a potential strategy for the modulation of dense ECM and enhancement of antibody efficacy.

## Introduction

Breast cancer is the most frequently diagnosed cancer and the second leading cause of cancer-related death among women worldwide (DeSantis et al., 2017). Overexpression of human epidermal growth factor receptor 2 (HER2) is detected in 20 to 30% of invasive breast cancers (Vogel et al., 2002; Hudis, 2007; Beyer et al., 2011). Therefore, HER2 targeted therapy has emerged as an effective strategy for the treatment of breast cancers in the clinic. Trastuzumab (Herceptin), a HER2-targeted monoclonal antibody (mAb), was one of the first monoclonal antibodies approved by FDA in 2011 (Shpilberg & Jackisch, 2013). It inhibits the proliferation and survival of HER2-positive tumors and has achieved great successes in the clinic (Shpilberg & Jackisch, 2013; Moslehi, 2016; von Minckwitz et al., 2017). However, the administration route of trastuzumab by intravenous (*i.v.*) infusion faces the several challenges, including complex operating processes, long infusion times, and infusion-related reactions. A Phase III clinical trial has been reported that co-delivery of trastuzumab with recombinant human hyaluronidase (rHuPH20) through subcutaneous (*s.c.*) administration achieved a comparable outcome to *i.v.* injection in terms of pharmacokinetic profiles, efficacy, and safety (Frost, 2007; Shpilberg and Jackisch, 2013; Xu et al., 2015). Therefore, localized delivery (e.g. *s.c.* injection) appears to be an alternative strategy for the administration of therapeutic antibodies.

The tumor microenvironment characterized by dense extracellular matrix (ECM) (most made of collagen-I) (Jain, 1990; Magzoub et al., 2008) and high interstitial fluid pressure (IFP) (Heldin et al., 2004), which reduce the transport and penetration of mAb in tumor (Jain and Stylianopoulos, 2010; Dewhirst & Secomb, 2017). After *i.v.* injection, accumulation of mAb was less than 0.01% of the injected dose per gram of tumor tissue with most circulating in the bloodstream (Marcucci et al., 2013; Shin et al., 2014). Some strategies have been developed to improve the penetration of biomacromolecules in solid tumors, such as manipulating the size, charge, and binding affinity of macromolecules, as well as coadministration of antitumor antibodies and collagenase or hyaluronidase (Netti et al., 2000; Shin et al., 2014; Xu et al., 2015). After *i.v.* injection of collagenase, IFP, and microvascular pressure (MVP) of solid tumor significantly decreased to 45 and 60%, respectively (Eikenes et al., 2004). As a result, the mAb accumulation in tumor tissue was dramatically increased (Eikenes et al., 2004). Therefore, coadministration of collagenase by a localized delivery system could be a potential strategy to enhance the penetration of antibody within stroma-rich solid tumors (e.g. breast cancers) (Provenzano et al., 2008; Visscher, 2011).

The thermosensitive hydrogel is a very promising localized delivery system and have gained great attention in the delivery of chemotherapeutics, peptide, and protein drugs (Klouda & Mikos, 2008; Lee et al., 2014; Lin et al., 2014; Shi et al., 2016). They have several advantages in drug administration, including ease of preparation and application, prolonged and localized drug delivery, low systemic toxicity, and good patient compliance (Ci et al., 2014; Lin et al., 2014). PLGA-PEG-PLGA triblock copolymer is one of the most widely exploited thermosensitive materials and has been widely developed as depot formulations for preclinical and clinical investigation (Cho & Kwon, 2014; Ci et al., 2014). The thermogels formed from PLGA-PEG-PLGA polymers showed a sustained release of loaded drugs for one week to several months *in vivo* due to the slow degradation of polyester (Wolinsky et al., 2012; Yu et al., 2013; Cho & Kwon, 2014; Ci et al., 2014; Chen et al., 2016).

**Figure 1.**
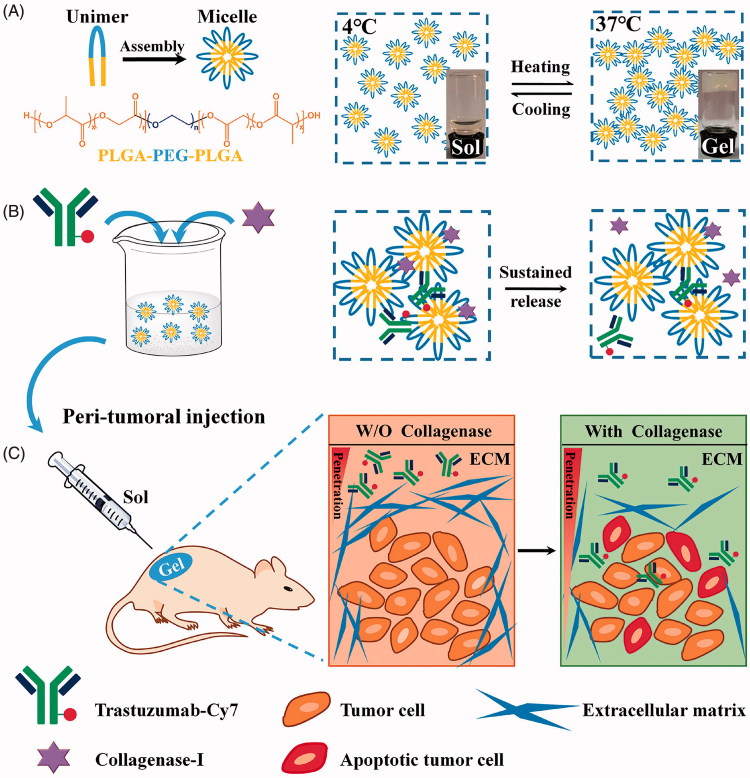
A schematic of the preparation of Col/Tra/Gel, which can degrade ECM and enhance penetration of therapeutic antibody in tumor. (A) The chemical structure of PLGA-PEG-PLGA triblock copolymer (left) and the sol–gel phase transition in water (right). (B) The preparation of thermosensitive hydrogels incorporated trastuzumab and collagenase-I. (C) The antitumor procedures of Col/Tra/Gel. After peritumoral injection, a drug-loaded biodegradable hydrogel will form in situ. Both collagenase and Cy7-trastuzumab will be slowly and sustainably released from the hydrogel. The dense ECM will be degraded by the released collagenase, followed by the deep penetration of trastuzumab into the tumor tissue, thereby inducing the tumor cell apoptosis.

Therefore, we hypothesized that co-delivery of trastuzumab and collagenase by an *in situ* thermosensitive hydrogel system can trigger the degradation of intratumoral collagen, promote drug penetration and retention, and finally enhance the antitumor efficacy (Figure 1).

Herein, the biodegradable PLGA-PEG-PLGA polymer was utilized to produce the injectable thermosensitive hydrogel system for the co-delivery of trastuzumab and collagenase. The hydrogels were characterized by thermosensitive properties, drug release, and stability *in vitro*. The peritumoral retention capacity, antitumor efficacy, and mechanism, as well as systemic toxicity were performed in stroma-rich BT474-xenografted BALB/c mice. Finally, the tissue reaction to the hydrogels was assessed in healthy BALB/c mice.

## Materials and methods

### Materials

PLGA-PEG-PLGA (MW: PEG = 1500 Da, MPLGA: MPEG = 3:1) was purchased from Shandong Daigang Inc. (Jinan, China). Collagenase-I was supplied by Invitrogen (Carlsbad, CA) and trastuzumab (Herceptin) was obtained from Roche (Basel, Switzerland). Cy7-NHS ester was obtained from GE Company (Boston, MA). Anti-CD31, anti-CD68, and anti-collagen antibodies were purchased from Abcam (Cambridge, UK). RPMI-1640, penicillin/streptomycin, and trypsin were purchased from M&C Gene Technology (Beijing, China). Fetal bovine serum (FBS) was from Gibco (Invitrogen, Carlsbad, CA). BT474 cells were from China Center for Type Culture Collection (Wuhan, China).

### Preparation of the trastuzumab-loaded hydrogel

The trastuzumab-loaded PLGA-PEG-PLGA hydrogel was prepared by the ‘cold’ method as previously reported (Lin et al., 2014; Hu et al., 2015). In brief, 2 g of PLGA-PEG-PLGA was dissolved in 8 ml of 0.9% NaCl solution and stirred at 4 °C until the polymer was dissolved completely and the clear solution was obtained. In the present experiment, the concentration of PLGA-PEG-PLGA polymer in the hydrogel was 20% (wt. %). To prepare the drug-loaded hydrogel, the trastuzumab and collagenase were carefully weighed and placed in a glass vial and then the cold blank hydrogel was added into the vial. The mixture was stirred at 4 °C overnight and the clear solution was formed. The prepared trastuzumab/PLGA-PEG-PLGA and collagenase/trastuzumab/PLGA-PEG-PLGA hydrogels were abbreviated as Tra/Gel and Col/Tra/Gel, respectively.

### *In vitro* characterization of thermosensitive hydrogel

The gel formation temperature (GFT) of blank hydrogel and Col/Tra/Gel was determined by the vial inverting method. The rheological properties of blank hydrogel were determined using a rheometer (MCR301; Anton Paar, Austria). The morphology of the blank hydrogel was visualized by Cryo-SEM (SU8010; Hitachi, Shiga, Japan). The samples were cryo-fixed by liquid nitrogen and sputtered with gold before analysis. Circular dichroism (CD) spectrum (190–240 nm) was to investigate the antibodies stability during storage. The release profiles of protein-loaded hydrogel were evaluated at 37 °C and measured by BCA method (Smith et al., 1985). All the details could be found in the Supplementary information.

### Animals and tumor model

Female nude mice (Nu/Nu, 18–20 g) were obtained from Vital River Laboratory Animal Center (Beijing, China) and were housed under SPF conditions. Tumor-bearing mice model was established by inoculating 1 × 106 BT474 cells in the flank. Tumors were allowed to reach a volume of ∼100 mm^3^ before treatment. All animal procedures were performed in accordance with the Guideline for Care and Use of Laboratory Animals of Peking University and approved by the Animal Ethics Committee of Peking University.

### Peritumoral retention of the trastuzumab-loaded hydrogel

The tumor-bearing mice were randomly divided into three groups that received *i.v.* injection of Cy7-conjugated trastuzumab (Cy7-Tra) solution, *s.c.* injection of Cy7-Tra/Gel, and Col/Cy7-Tra/Gel hydrogels, respectively. After treatment, mice were anesthetized with 2% isoflurane and the near-infrared (NIR) images were captured by Kodak In Vivo Imaging System (Carestream Health, ‎Rochester, NY) at 0, 1, 2, 3, 4, 5, 13, 18, 20 days post-injection. Then, the mice were sacrificed by cervical dislocation. The tumors and major organs were collected and imaged with the same system.

### *In vivo* antitumor efficacy

BT474 tumor-bearing mice were randomly divided into seven groups with six mice in each group. Each group of mice was treated with PBS, blank gel, trastuzumab/Gel (Tra/Gel), collagenase/trastuzumab/Gel (Col/Tra/Gel), hyaluronidase/trastuzumab/Gel (Hya/Tra/Gel), trastuzumab solution for single *i.v.* injection (Tra–Sol single *i.v.*), and trastuzumab solution for multiple *i.v.* injection (Tra–Sol multiple *i.v.*), respectively. All the hydrogel formulations were subcutaneously injected into the peritumoral area of the mice for only once. Tra–Sol single *i.v.* and Tra–Sol multiple *i.v.* groups were intravenously injected for one and four times with 7.5 mg/kg/week, respectively. The overall dosage of trastuzumab in each group was 30 mg/kg. The tumor size was calculated by the equation: V (mm^3^) = [length × (width)^2^]/2. The body weight of mice was recorded every five days.

In a separate experiment, a total of 12 mice were divided into two groups. Each group of mice was received single *s.c.* injection of high dose of Col/Tra/Gel (30 mg/kg) or low dose of Col/Tra/Gel (10 mg/kg). The tumor size and body weight of mice were recoded.

### Comparison with clinical treatment regimes

BT474 tumor-bearing mice were injected with different trastuzumab-loaded formulations. Clinical formulations, including Hya/Tra *s.c.* (trastuzumab 10 mg/kg, hyaluronidase 20 mg/ml) and Tra–Sol *i.v.* (trastuzumab 10 mg/kg) were injected weekly for four times with a total dosage of 40 mg/kg of trastuzumab. Tra/Gel *s.c.* (trastuzumab 10 mg/kg), Col/Tra/Gel *s.c.* (trastuzumab 10 mg/kg, Collagenase 20 mg/ml), and Col/Gel *s.c.* (collagenase 20 mg/ml) were injected for once with a total dosage of 10 mg/kg of trastuzumab.

### TUNEL assay and immunohistochemistry

At the end of the antitumor study, the mice were sacrificed and major organs, as well as tumors were collected. The tumor tissues were frozen for cell apoptosis, collagen, and CD31 staining. For collagen staining, sections were fixed, blocked, and incubated with rabbit anti-mouse collagen antibody (1:50) at 4 °C overnight followed by incubating with Alexa Fluor 647-conjugated secondary antibody (1:100) for 1 h at room temperature. For TUNEL analysis, experiments were carried out according to manufacturer’s standard protocol. For the immunohistochemistry of CD31, sections were fixed, blocked, and incubated with rabbit anti-mouse CD31 antibody (1:50) at 4 °C overnight followed by incubating with the FITC-conjugated secondary antibody (1:100) for 1 h at room temperature. Cell nuclei were stained with DAPI and the sections were covered with a coverslip. The sections were scanned by Leica fluorescence microscope system. All the images were captured at ×200 magnification. The major organs, including heart, liver, lung, and kidney were fixed in 10% formalin for H&E staining.

### Tissue reaction

A total of 30 healthy BALB/c mice were randomly divided into five groups with six mice in each group. Each group of mice was treated with 5% glucose, blank gel, Col/Gel, Hya/Gel, respectively. After 6 months posttreatment, the mice were sacrificed and tissues of injected sites were collected and fixed in 10% formalin for H&E and CD68 staining.

### Statistical analyses

Data were presented as the mean ± standard deviation (SD). Student’s *t*-test was used for all statistical analyses between two groups. Multiple comparisons were performed using a one-way analysis of variance (ANOVA). The values of *p* less than .05 or .01 were considered to be significant and highly significant (*p < .05, **p < .01).

## Results

### Characterization of the thermosensitive hydrogel

**Figure 2.**
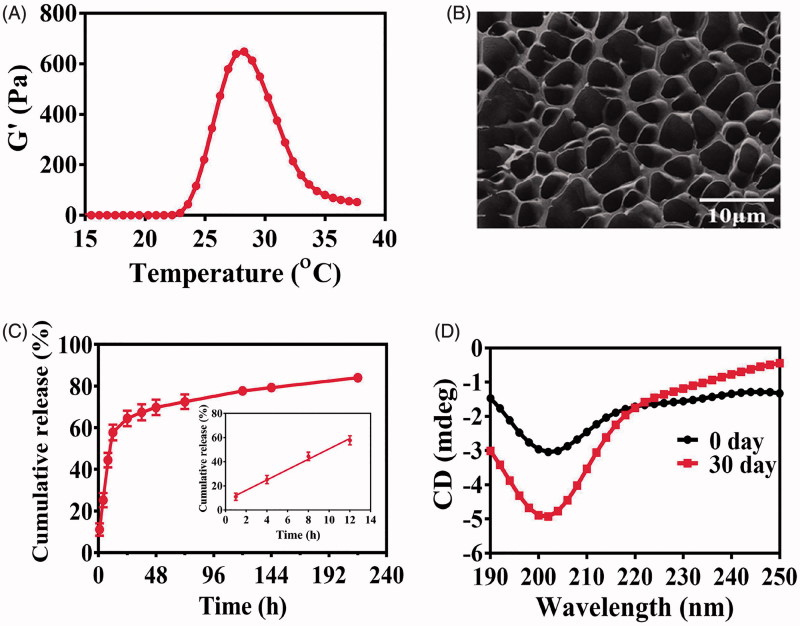
Characterization of the thermosensitive hydrogel. (A) Storage modulus (*G′*) of the blank PLGA-PEG-PLGA hydrogel. (B) The SEM imaging of blank PLGA-PEG-PLGA hydrogel (scale bar =10 μm). (C) Release profiles of protein from the hydrogel. Inset is the release profile of the first 12 hours. (D) Storage stability of Col/Tra/Gel in 0 and 30 days at 4 °C observed by the CD spectrogram.

The blank PLGA-PEG-PLGA hydrogel was a transparent liquid below 15 °C that allowed for the good syringeability. The viscosity of the polymer solution increased sharply with the temperature rising, and finally, the liquid turned into opalescent solid at 37 °C (Figure 1(A)). The blank gel displayed a reversible sol–gel transition behavior. The GFT of the blank hydrogel and Col/Gel was 27.27 ± 0.25 and 26.03 ± 0.25 °C, respectively, without significant difference (*P* > .05), indicating that the co-loading of collagenase did not change the thermosensitive properties of hydrogels (Figure 2(A)). When the temperature increased to ∼27–28 °C, the shear stress and storage modulus expanded steeply that was consistent with the GFT results (Figure S1). The Cryo-SEM images showed that the microstructure of the hydrogel is a three-dimensional porous structure (Figure 2(B)). The meshes make it possible for the encapsulation of the biomacromolecules, such as collagenase or trastuzumab. Because of the complexity of the meshes, the encapsulated biomacromolecules could be retained within the hydrogel matrix and minimize the burst release.

### *In vitro* drug release and stability

The cumulative release profile of trastuzumab from the Tra/Gel hydrogel at 37 °C was illustrated in Figure 2(C). More than 85% of incorporated proteins were released from the hydrogel within 9 days. The release profile is biphasic, with a relatively fast release at the first 12 hours followed by a sustained release until the 9th day. This rapid release of trastuzumab in the first phase accounts for nearly 60% of cumulative release and could be attributed to large mesh sizes of the hydrogel, which allows for the zero-order release of trastuzumab from the gel matrix. A sustained release in the second phase accounts for more than 25% of total released drugs over 9 days.

The CD spectrum is a classical method to monitor the structure changes of biomacromolecules and evaluate the biological activity. In the far UV region (190–240 nm), a peptide bond is the main chromophore, from which the secondary structures such as α-helix and β-sheet can be analyzed to illustrate the changes of protein structures. CD spectra showed that the structures of hyaluronidase, collagenase, and trastuzumab in aqueous solution did not change apparently during 30 days of storage at 4 °C (Figure S2). However, some changes in the CD spectrum of PLGA-PEG-PLGA polymer solution were found that probably because of the degradation of the ester bond of the polymer. As shown in Figure 2(D), the CD spectrum of Col/Tra/Gel at 0 day is the superimposition of the individual curve of PLGA-PEG-PLGA polymer, collagenase, and trastuzumab. After 30 days storage, the CD curve of Col/Tra/Gel has changed apparently. These changes in terms of the shape and peak position were consistent with those of PLGA-PEG-PLGA polymer solution. Thus, the difference in CD spectra of Col/Tra/Gel between 0 and 30 days could be attributed to the degradation of PLGA-PEG-PLGA polymer. These results suggested that the incorporated proteins kept stable during 30 days of storage.

### *In vivo* long-term retention by NIR imaging

**Figure 3.**
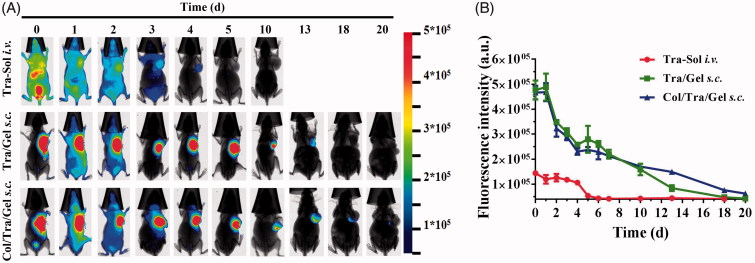
*In vivo* sustained drug release by NIR imaging. (A) *In vivo* NIR images of BT474 tumor-bearing mice after *i.v.* injection of Cy7-Tra solution (upper) and *s.c.* injection of Cy7-Tra/Gel (middle) and Col/Cy7-Tra/Gel (lower) at 0, 1, 2, 4, 5, 7, 13, 18, 20 days (*n* = 3). (B) Quantification of the fluorescence intensity of *in vivo* NIR images as a function of time post-injection of Tra–Sol *i.v.* (red), Tra/Gel *s.c.* (green), and Col/Tra/Gel *s.c.* (blue) (*n* = 3).

To track the retention of thermosensitive hydrogels *in vivo*, an NIR dye, Cy7 was conjugated to the trastuzumab through NHS ester reaction with a primary amine. Figure 3 illustrated a series of NIR fluorescence images of BT474 tumor-bearing mice at 0, 1, 2, 3, 4, 5, 10, 13, 15, 18, and 20 days after treatment with different groups, including Tra–Sol *i.v.*, Tra/Gel, *s.c.*, and Col/Tra/Gel, *s.c.* All of the three groups exhibited the NIR signals slowly decayed over time. In the Tra–Sol *i.v*. group, the antibody was distributed throughout the whole body after *i.v.* injection. The injected antibody was accumulated in tumor site at 1 day post-injection and rapidly eliminated within 4 days. Compared with the Tra–Sol *i.v.* group, the fluorescence signals of Tra/Gel *s.c.* and Col/Tra/Gel, *s.c.* groups could retain for 13 and 20 days, respectively. The gel system presented much longer retention compared with the traditional *i.v.* route. We also found that the Col/Tra/Gel, *s.c.* group has longer retention time than the Tra/Gel *s.c.* group. As shown in Figure 3(B), the quantitative fluorescence intensity of *in vivo* NIR imaging verified the long-term release of trastuzumab from Tra/Gel *s.c.* and Col/Tra/Gel *s.c.* without obvious initial burst release. These results illustrated that collagenase degraded the collagen in the tumor, making the tumor structure loose and enhancing the penetration of trastuzumab within the tumor, thereby resulting in the extended tumor retention.

### Antitumor efficacy *in vivo*

**Figure 4.**
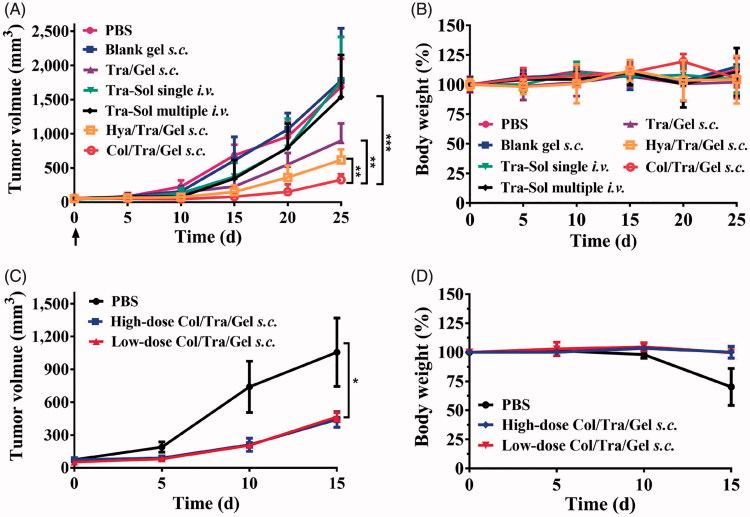
Anti-tumor efficacy and body weight change. Inhibition of tumor growth (A) and body weight (B) in BT474 tumor-bearing mice after treatment with different formulations at a total dose of 30 mg/kg of trastuzumab (*n* = 6). The tumor growth (C) and body weight (D) in BT474 tumor-bearing mice after treatment with Col/Tra/Gel at high dose (30 mg/kg) or low dose (10 mg/kg).

The anticancer effects of Col/Tra/Gel were evaluated in HER2-positive BT474 tumor-bearing mice. The Col/Tra/Gel treatment exhibited the greatest inhibition of tumor growth during the whole experiments (Figure 4(A)). All of the drug-loaded hydrogels formulations, including Tra/Gel *s.c.*, Col/Tra/Gel *s.c.*, and Hya/Tra/Gel *s.c.* groups demonstrated significant inhibitory effects against the tumors compared with the other groups, including PBS, Blank gel *s.c.*, Tra–Sol single *i.v.*, and Tra–Sol multiple *i.v.* (**P < .01). Tra–Sol single *i.v.* and Tra–Sol multiple *i.v.* groups both achieved good inhibitory effects in the early 15 days, whereas these two groups did not demonstrate any significant inhibition of tumor growth in the end that could be due to the fast elimination of the trastuzumab by *i.v.* injection. In addition, mice of all groups maintained their normal weight throughout the experiments (Figure 4(B)). These results demonstrated that the *s.c.* treatment of Col/Tra/Gel enhanced the antitumor efficacy of trastuzumab.

To investigate the dose-dependent antitumor efficacy, BT474 tumor-bearing mice were treated with 10 and 30 mg/kg of the Col/Tra/Gel hydrogels (on the basis of trastuzumab), respectively. As showed in Figure 4(C), both high-dose and low-dose treatments demonstrated the comparable inhibitory effect on tumor growth with negligible body weight changes (Figure 4(D)). This result demonstrated that the Col/Tra/Gel treatment at low dose could achieve the identical therapeutic effect to the high-dose regime, suggesting Col/Tra/Gel treatment at low dose is a cost-effective strategy for the treatment of breast cancers.

**Figure 5.**
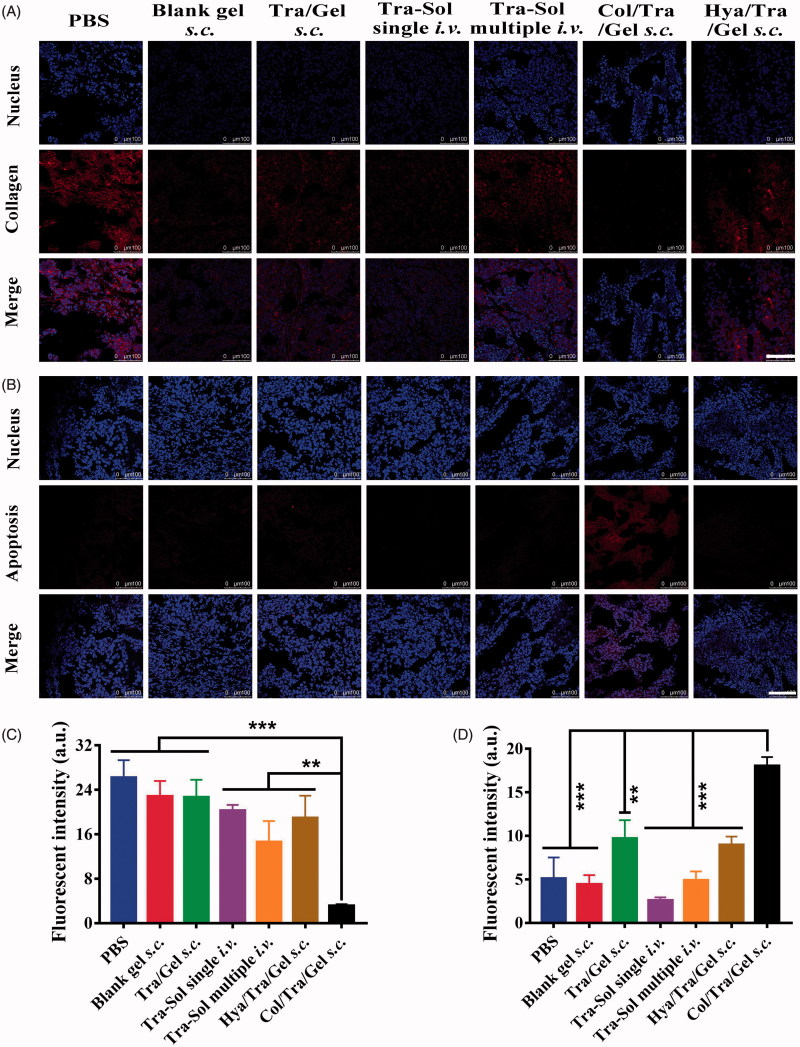
*In vivo* collagen degradation and apoptosis staining. (A) Degradation of collagen in tumor tissues was analyzed by immunohistochemistry (red, collagen; blue, nuclei; scale bar, 100 µm). (B) Cell apoptosis in tumor tissues was analyzed by TUNEL assay (red, apoptotic cells; blue, nuclei; scale bar, 100 µm). (C) Quantitative assay of collagen staining. (D) Quantitative assay of apoptotic staining. *p < .05; **p < .01; ***p < .001, compared with the Col/Tra/Gel *s.c.* group.

The tumor tissues were further analyzed by immunohistochemistry (Figure 5). The collagen, TUNEL, and CD31 assays were performed to evaluate the collagen density, apoptotic cells, and tumor vessel density, respectively. Collagen assays showed that Col/Tra/Gel treatment resulted in the lowest density of collagen compared with other groups (Figure 5(A)). The quantitative results of collagen staining were presented in Figure 5(C). The collagen density of the Col/Tra/Gel group was 4 times lower than that of the Tra–Sol -*i.v.* single group, demonstrating that collagenase-I could degrade the collagen in the ECM and promote the antibodies penetrated into the deep tumor tissues. TUNEL assays revealed that the Col/Tra/Gel group exhibited the most significant apoptotic effect in BT474 tumor cells, whereas Tra/Gel and other groups did not present any obvious apoptotic cells (Figure 5(B)). The percentage of TUNEL positive cells in the Col/Tra/Gel group was 4-fold higher than that of the Tra–Sol multiple *i.v.* group and over 6-fold higher than that of the Tra–Sol single *i.v.* group (Figure 5(D)). CD31 is a biomarker of tumor vasculature related to the angiogenesis. CD31 images further demonstrated that the Col/Tra/Gel group exhibited the strongest anti-vasculature effect, whereas Tra/Gel did not induce any obvious anti-vasculature effect (Figure S3). The density of tumor vessels in the PBS group was 9-fold higher than that of the Col/Tra/Gel group. These results proved that the Col/Tra/Gel treatment induced the degradation of collagen and apoptosis of tumor cells, further confirming the essential of collagenase for the degradation of collagen and the deep penetration of trastuzumab for enhanced antitumor efficacy.

The major organs were analyzed by H&E staining. As shown in Figure S4, no obvious change was found in all of the excised organs from the Col/Tra/Gel group. However, the PBS group showed visible tumor metastasis in liver. Significant trastuzumab-related cardiotoxicity was found in the heart tissues of Tra–Sol single *i.v.* and Tra–Sol multiple *i.v.* groups. In addition, the lung metastasis was observed in Tra–Sol multiple *i.v.* groups. These results verified that *s.c.* treatment of Col/Tra/Gel formulation reduces the possibility of side effects caused by trastuzumab.

### Comparison with clinical treatment regimes

**Figure 6.**
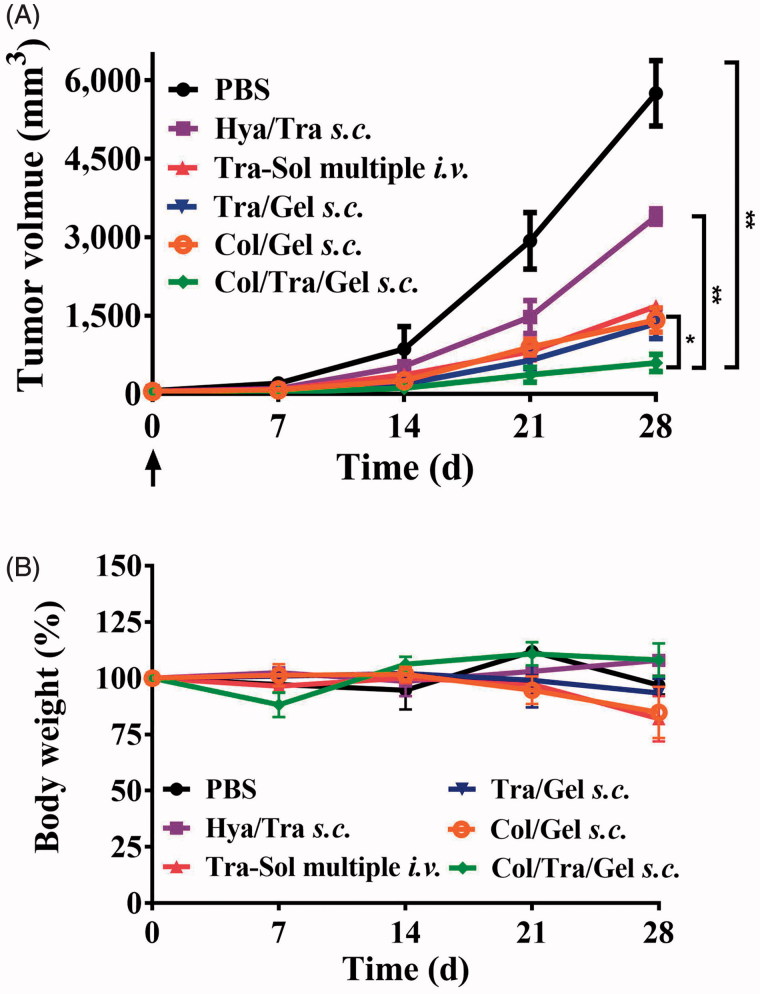
Comparison of the antitumor efficacy of Col/Tra/Gel with different clinical regimes in BT474 tumor-bearing mice. The tumor growth (A) and body weight (B) in BT474 tumor-bearing mice after treatment with different formulations (*n* = 6). *p < .05; **p < .01; ***p < .001, compared with the Col/Tra/Gel *s.c.* group.

The anti-cancer effects of Col/Tra/Gel were also compared with the clinical regimes, including Hya/Tra *s.c.* (trastuzumab 10 mg/kg, Hyaluronidase 20 mg/ml) injected only once and Tra–Sol *i.v.* (trastuzumab 10 mg/kg) injected once a week with a total dosage of 40 mg/kg of trastuzumab. Figure 6 showed that the Col/Tra/Gel group (total dosage of 10 mg/kg of trastuzumab) achieved the greatest suppression of tumor growth. Other groups including Col/Gel *s.c.*, Tra/Gel, and Tra–Sol *i.v.* displayed comparable antitumor effect, whereas Hya/Tra *s.c.* showed the worst antitumor efficacy. No significant body weight change was found in all of the treatment groups.

### Biosafety of thermosensitive hydrogels

To check whether the *s.c.* injection of Col/Tra/Gel would induce the inflammation, the macrophage recruitment in the injected sites was analyzed by CD68 immunohistochemistry. The yellow zone is an indication of the inflammation areas. As shown in Figure S5, no obvious inflammatory response was found in all of the hydrogel groups, including blank gel, Col/Gel, Hya/Gel, and Col/Tra/Gel at 6 months posttreatment. In addition, H&E images of injected sites did not show obvious pathophysiological changes as compared to the PBS group (Figure S5). These results suggested that the thermosensitive hydrogel had good biocompatibility.

## Discussion

Monoclonal antibodies (mAbs) have become a mainstay of targeted cancer therapy. However, the poor accumulation and penetration of mAbs in solid tumors caused by the dense ECM limited their antitumor efficacy (Minchinton & Tannock, 2006). It has been demonstrated that modulation of the tumor ECM improved the interstitial transport and cellular uptake of antibodies. Several strategies have been developed to modulate the dense ECM, decrease the high IFP, enhance interstitial transport of antibodies, and finally enhance the antitumor efficacy of antibodies (Jain, 1990; Minchinton & Tannock, 2006; Kato et al., 2012; Marcucci et al., 2013; Dewhirst & Secomb, 2017). ECM-degrading enzymes, including collagenase and hyaluronidase, have been exploited to digest the collagen and hyaluronan, respectively, by *i.v.*, *s.c.*, or intratumoral injections (Kato et al., 2012; Marcucci et al., 2013). It has been reported that the collagenase-induced reduction in IFP and MVP can only maintain from 100 min to several hours probably due to the rapid clearance of collagenase from the body. Thus, localized co-delivery of collagenase and trastuzumab will be essential to maintain the loose ECM and enhance the penetration and retention of trastuzumab in solid tumors for a prolonged time.

Here, we developed the Col/Tra/Gel system, which was a transparent liquid at 4 °C and can form semi-solid hydrogel at body temperature at injected sites (Figure 1(A)). The viscosity of the Col/Tra/Gel is very low and makes it easy to pass through the syringe needle at low temperature. After peritumoral injection, the hydrogel was formed quickly to control release of two protein drugs. Compared with Pluronic F127 hydrogel, the PLGA-PEG-PLGA thermosensitive hydrogel showed stronger mechanical integrity, higher stability, and slower dissolution (Klouda & Mikos, 2008). This may be due to the slow degradation of the PLGA-PEG-PLGA copolymer, as well as hydrophilic PEG as a cross-linking agent and PLGA polymer chains shrinking as hydrophobic core (Ghahremankhani et al., 2008, Sood et al., 2016). As shown in Figure 2(C), the Col/Tra/Gel system presented a relatively fast release through zero-order kinetics within the first 12 hours followed by a sustained release until the 9 day. The total released proteins accounts for over 85% of the total amount of proteins. The fast released phase could induce the digestion of the collagen at the first several hours posttreatment, and enhance the localization and penetration of trastuzumab in solid tumors. The sustained release phase will probably maintain the loose ECM and allow for the accumulation and penetration of trastuzumab for long period of time. The *in vivo* imaging results have demonstrated that the retention of trastuzumab in the Col/Tra/Gel *s.c.* group lasted for 20 days, whereas those of Tra–Sol *i.v.* and Tra/Gel *s.c.* groups only maintain for 4 and 13 days, respectively (Figure 3). These results also indicated that trastuzumab was released from hydrogel *in vivo* in a sustained manner without obvious initial burst release. The longer retention of trastuzumab could be attributed to the biphasic and prolonged release of collagenase and trastuzumab from Col/Tra/Gel system.

As shown in Figure 4(A), all the drug-loaded hydrogel groups through *s.c.* injection demonstrated significant inhibitory effects on the tumors compared with trastuzumab solution through single or multiple *i.v.* injection. As hyaluronic acid is also an important component of the ECM in solid tumors, the antitumor effect of Hya/Tra/Gel was also investigated. Results illustrated a remarkable tumor inhibition after treatment with Hya/Tra/Gel *s.c.*, which is consistent with the findings reported by other groups (Xu et al., 2015). Compared with Tra/Gel, Hya/Tra/Gel, and other groups, the Col/Tra/Gel exhibited the greatest inhibitory effect on tumor growth. The immunohistochemistry results further demonstrated that the collagen was significantly digested and increased apoptotic cell death was found in the tumor tissues after treatment with Col/Tra/Gel formulation (Figure 5). These results demonstrated that treatment with Col/Tra/Gel *s.c.* could digest the collagen, enhance the retention and uptake of trastuzumab in tumor tissues, thereby augment the antitumor efficacy of trastuzumab, which is correlated well with the results of *in vivo* imaging.

As illustrated in Figure 4(C), low-dose (10 mg/kg of trastuzumab) of Col/Tra/Gel could achieve comparable antitumor efficacy to high-dose (30 mg/kg of trastuzumab) of Col/Tra/Gel. Unexpectedly, single *s.c.* injection of Col/Tra/Gel at a quarter-dose (10 mg/kg of trastuzumab) successfully achieved the better suppression of tumor growth compared with *i.v.* injection of clinical trastuzumab formulation for 4 times with a total dose of 40 mg/kg (Figure 6). In addition, the antitumor efficacy of Tra–Sol multiple *i.v.* would be enhanced when the total injected dose was increased from 30 to 40 mg/kg (Figures 4 and 6) Heart failure is a typical side effect of trastuzumab in the clinic (Vogel et al., 2002). Histological analysis revealed that no cardiotoxicity was found in the heart tissues of Col/Tra/Gel, whereas significant side effects were observed in Tra–Sol single *i.v.* and Tra–Sol multiple *i.v.* groups. Moreover, no obvious inflammatory response and systemic toxicity were found in all of the hydrogel groups at 6 months posttreatment (Figure S5). These results proved that the Col/Tra/Gel *s.c.* offers a convenient, cost-effective, biocompatible, and efficient strategy for HER2 positive cancer therapy.

## Conclusions

We developed a Col/Tra/Gel system for co-delivery of trastuzumab and collagenase. The Col/Tra/Gel showed the continuous and biphasic release of protein drugs for 9 days *in vitro*. Col/Tra/Gel exhibited longer localized retention time than Tra/Gel and Tra–Sol groups. The *s.c.* treatment of Col/Tra/Gel resulted in the greatest inhibition of tumor growth compared with other groups *in vivo.* Moreover, quarter-dose of Col/Tra/Gel obtained a better antitumor efficacy compared with the *i.v.* injection of trastuzumab solution. Histological and immunohistochemical analyses proved that the Col/Tra/Gel treatment induced the digestion of collagen and apoptotic cell death. In addition, the Col/Tra/Gel incurred a low tissue reaction and system toxicity. Therefore, we have demonstrated that a Col/Tra/Gel system for breast cancer therapy that triggered the degradation of intra-tumoral collagen, promote penetration and retention, and finally enhance the antitumor efficacy of trastuzumab. We believed that this localized co-delivery system offers a potential strategy for the modulation of dense ECM and enhancement of antibody efficacy.

## Supplementary Material

Supporting Information
