# Supplementary material for: Localized co-delivery of collagenase and trastuzumab by thermosensitive hydrogels for enhanced antitumor efficacy in human breast xenograft
Source: Drug Deliv. 2018 Jun 26;25(1):1495–503. doi: 10.1080/10717544.2018.1474971 (PMC6058501; doi:10.1080/10717544.2018.1474971)
Supplement: Supporting Information [file IDRD_A_1474971_SM0147.docx]

**Supporting information**

**Localized co-delivery of collagenase and trastuzumab by thermosensitive hydrogels for enhanced antitumor efficacy in human breast xenograft**

Anni Pan^1,2,#^, Zhaoyang Wang^2,#^, Binlong Chen^1,2^, Wenbing Dai^2^, Hua Zhang^2^, Bing He^2^, Xueqing Wang^2^, Yiguang Wang^1,2,^*, Qiang Zhang^1,2,^*

1. State Key Laboratory of Natural and Biomimetic Drugs, School of Pharmaceutical Sciences, Peking University, Beijing 100191, China;

2. Beijing Key Laboratory of Molecular Pharmaceutics, School of Pharmaceutical Sciences, Peking University, Beijing 100191, China.

**#** These authors contributed equally to this work.

Correspondence should be addressed to Qiang Zhang ([zqdodo@bjmu.edu.cn](mailto:zqdodo@bjmu.edu.cn)) and Yiguang Wang ([yiguang.wang@pku.edu.cn](mailto:yiguang.wang@pku.edu.cn))

**Supporting Methods**

**Gel formation temperature (GFT) of copolymer solution.**

The solution of PLGA-PEG-PLGA blank gel was prepared and sealed in a small sample vial. The blank gel was heated slowly from 10 °C to 30 °C at a rate of 0.5 °C/min controlled by a temperature control system. The sample was equilibrated for 15 min at each temperature point, and the flowability of the sample was monitored by tilting the vials. The gel formation temperature (GFT) was recorded when the liquid was turned into immobile gel.

**Rheological studies**

The rheological behavior of the PLGA-PEG-PLGA blank gel was investigated by measuring the temperature-dependent viscosity curve using a digital viscometer. The polymer solution in ice-bath was placed on parallel plates. The temperature was controlled at a heating rate of 1 ºC/min with a temperature precision of ±0.1 ºC. The sample was equilibrated for 15 min at each temperature point before measurement.

**Stability study**

The stability of hydrogel components during storage was investigated using circular dichroism (CD) spectroscopy. The trastuzumab solution (1.5 mg/ml), collagenase solution (1.5 mg/ml), blank hydrogel, and Col/Tra/Gel (trastuzumab 4 mg/ml, collagenase 20 mg/ml) were stored at 4 ºC for 30 days, respectively and then the samples were analyzed by CD spectrum (190-260 nm).

***In vitro* release of trastuzumab from hydrogel**

A volume of 300 μl of Tra/Gel at 4 ºC was added into the transwell insert and placed into a water bath (37 ºC) to allow for the formation of clear gel. Then, the insert was placed into a 50 mL of tube containing 10 ml of normal saline (0.9% NaCl) pre-warmed to 37 ºC. The tube was placed in a thermostatic shaker under 37 ºC at 100 rpm. At pre-designated time points, the release medium (200 μl) was withdrawn and same volume of fresh medium was added. The concentration of trastuzumab in the release medium was measure by BCA protein assay.

**Synthesis of Cy7-Trastuzumab**

The trastuzumab and Cy7-NHS ester at a molar ratio of 1:10 was placed in 2 ml PBS, then 600 μl of 1 mol/l Na_2_HPO_4_ solution was added to adjust the pH to 8.5. The mixture was stirred for 15 min in the dark. The synthesized Cy7-Trastuzumab was purified by dialysis (MWCO=14000 Da) and the purified conjugate was lyophilized for storage and further investigation.

**Supplementary Figures:**

**
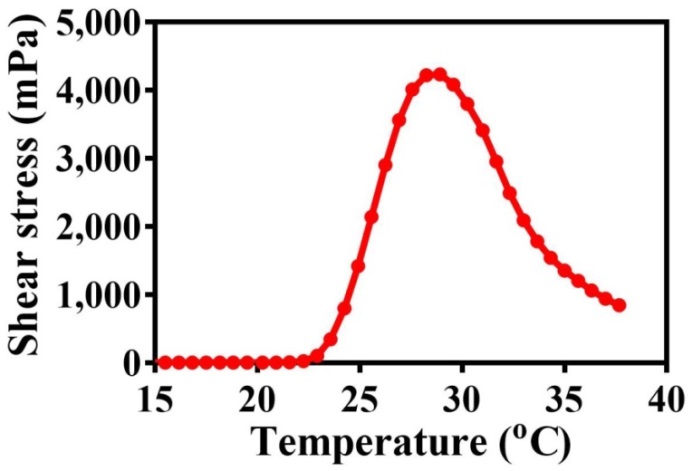
**

**Figure S1.** The rheology of blank PLGA-PEG-PLGA hydrogel.

**
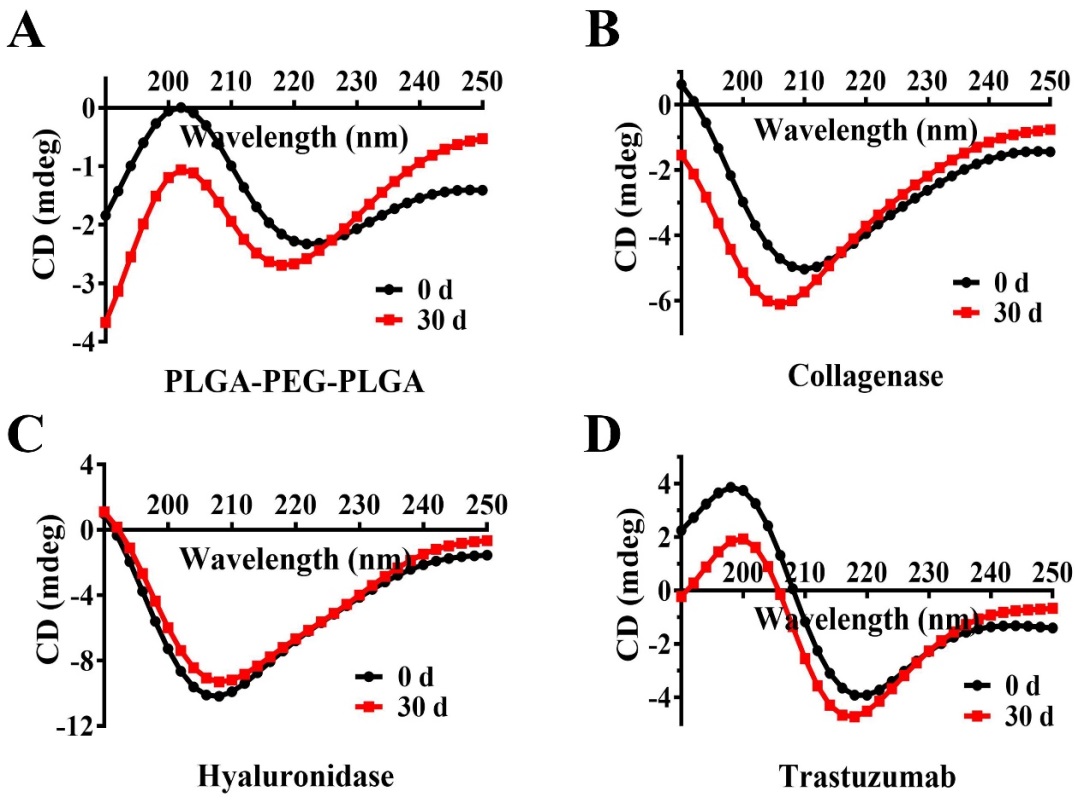
**

**Figure S2.** Stability of different components was measured by CD spectrogram. The components including: (A) blank PLGA-PEG-PLGA hydrogel, (B) collagenase solution (1.5 mg/ml), (C) hyaluronidase solution (1.5 mg/ml), and (D) trastuzumab solution (1.5 mg/ml).


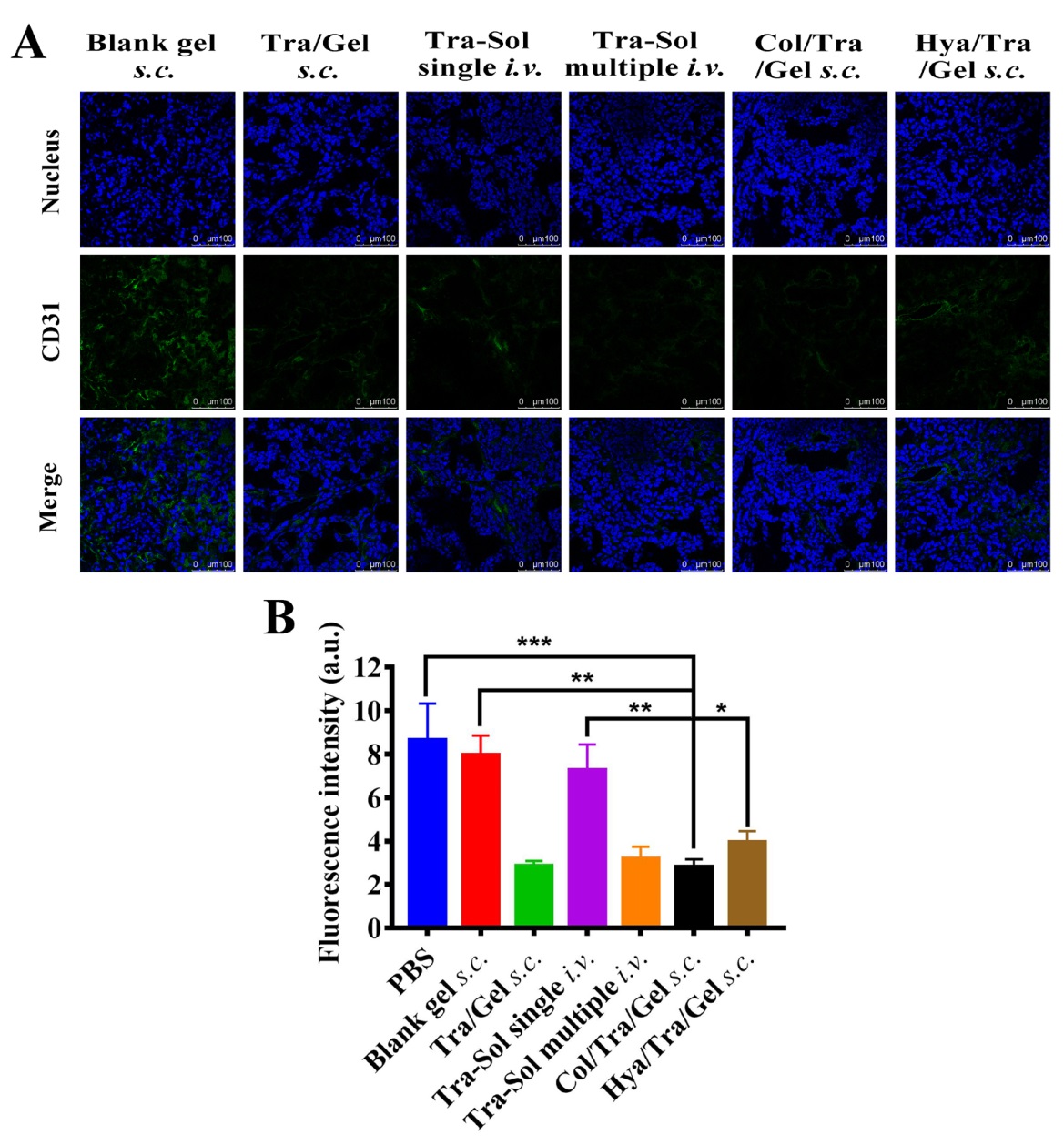


**Figure S3.** (A) Vasculature staining (CD31) of tumor tissues from BT474-inoculated mice after treatment with different formulations (green, blood vessels; blue, nuclei). Scale bar is 100 µm. (B) Quantitative assay of the CD31 staining. *, p<0.01; **, p<0.01; ***, p < 0.001, compared with Col/Tra/Gel *s.c.* group.


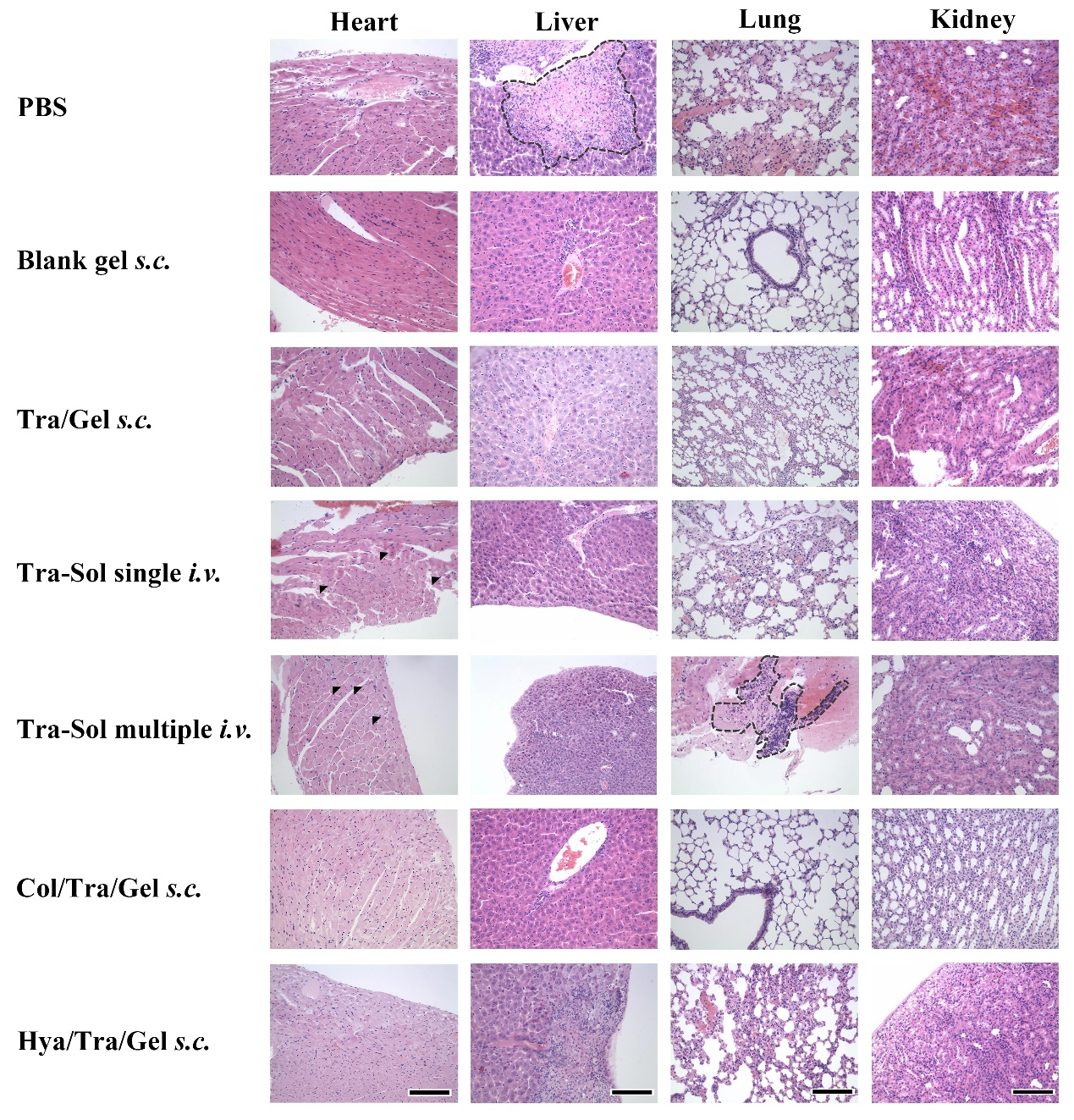


**Figure. S4** H&E staining of major organs collected from BT474-inoculated mice. The mice were treated with different formulations at a total dose of 30 mg/kg of trastuzumab. Scale bar is 100 μm.


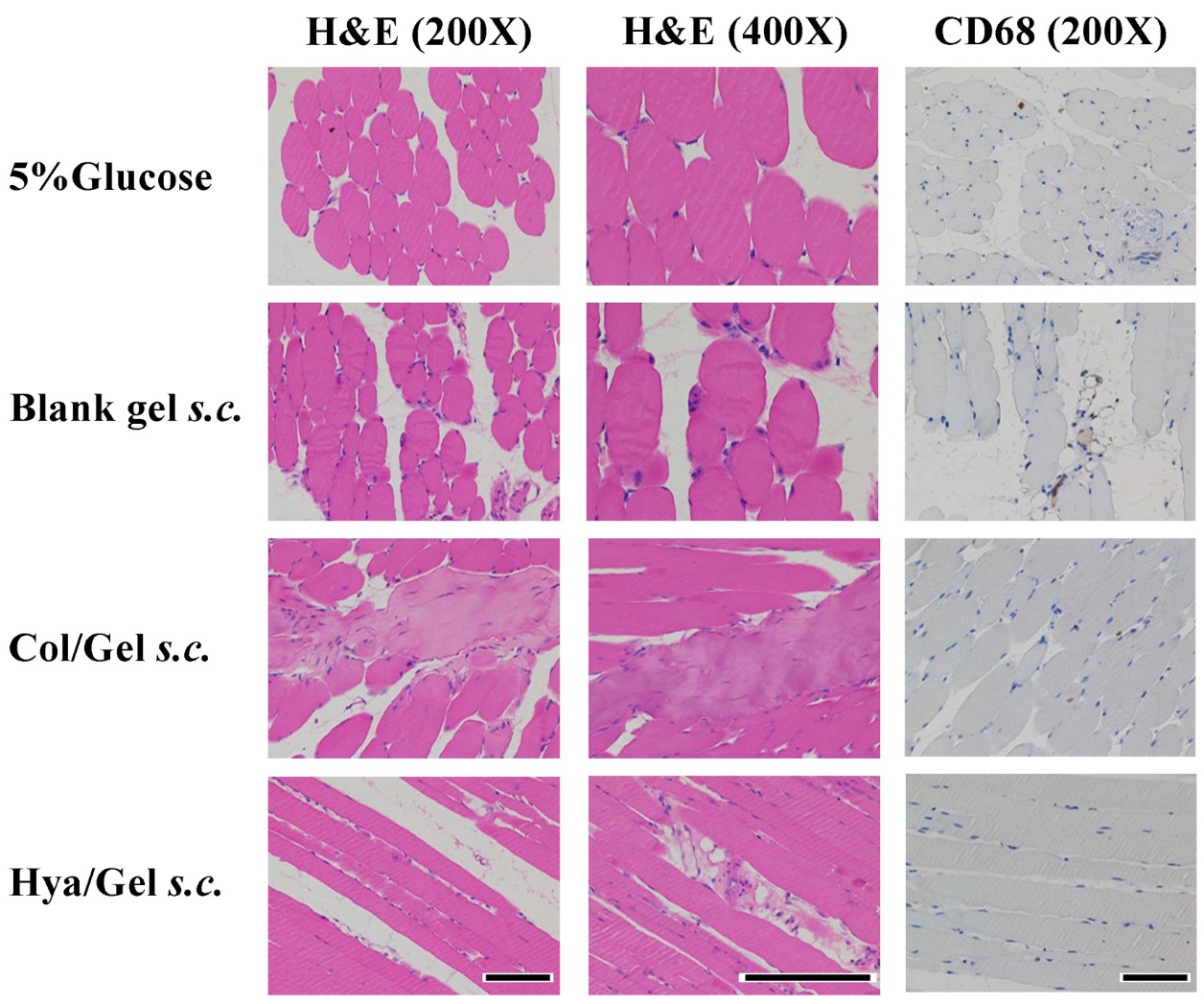


**Figure. S5** Histological (H&E) and immunohistochemical (CD68) analyses of injected sites from healthy mice. The mice were treated with 5% glucose, blank gel, Col/Gel, Hya/Gel, Hya/Col/Gel, respectively. Scale bar is 100 μm.
